# The multilevel influence of supervisor helping behavior on employee voice behavior: A moderated mediation model

**DOI:** 10.3389/fpsyg.2022.955288

**Published:** 2022-08-26

**Authors:** Peihua Fan, Yuzhao Liu, Haowen Liu, Mingjun Hou

**Affiliations:** ^1^School of Business and Management, Shanghai International Studies University, Shanghai, China; ^2^Department of Human Resources, NIO Inc., Shanghai, China; ^3^School of Business Administration, Shanghai Lixin University of Accounting and Finance, Shanghai, China

**Keywords:** supervisor helping behavior, voice behavior, thriving at work, psychological availability, experience sampling method

## Abstract

Based on conservation of resource theory, this study adopts an experience sampling method to build a cross-hierarchical mode to investigate the internal mechanism between supervisor helping behavior and employee voice behavior. The empirical results from 76 employees’ dynamic data show that the supervisor helping behavior has no significant direct effect on the employee voice behavior; thriving at work plays a mediating role between supervisor helping behavior and employee voice behavior. Psychological availability, as a moderator, not only positively moderates the effect of supervisor helping behavior on thriving at work but also positively moderates the mediation of thriving at work on the relationship between supervisor helping behavior and employee voice behavior. From the dynamic perspective, this study adds to the literature on supervisor helping behavior and employee voice behavior, and it has practical implications on managerial decision-making.

## Introduction

The intensification of market competition prompts enterprises to engage in innovation and reform and continuously improve through internal innovation and external learning to gain a competitive advantage (Ardito et al., 2021). Some enterprises place a high value on employee opinions and encourage them to contribute their knowledge and strength to the organization and improve the organizational effectiveness (Hsiung, 2012). China Shenyang Blower Works Group Corporation has publicly displayed outstanding examples of bottom-tier employees’ improvement proposals and voices since 2020, including heat treatment craft improvement, flange placement scheme optimization, and the improvement of machining mode and measuring tools. The various innovation suggestions made by employees provide strong support for enterprise technological innovation.

Meanwhile, the necessity of internal communication channels and employees’ voices has also been repeatedly emphasized in existing literature (Ewing et al., 2019; Yue et al., 2021). Voice behavior, from a “bottom-up” perspective, can both stimulate employees’ responsibility and organizational citizen behavior and form the management problem diagnosis system, improve the working environment, and boost core competitiveness (Tedone and Bruk-Lee, 2021). “Voice behavior” refers to an extra-role behavior in which employees spontaneously share constructive suggestions with their superiors and point out work problems and decision-making mistakes to improve organizational performance (Ran and Zhou, 2020). The current literature has primarily discussed the driving factors of voice behavior from the internal employee individual and external organizational environment, including social responsibility, personality traits, work values, job satisfaction, organizational justice, and leadership style (Liu et al., 2021). In addition to leadership styles and characteristics, scholars are paying attention to the impact of daily management behaviors such as supervisor helping behavior toward employees (Son, 2019). Leadership trust is closely related to employees’ extra-role behavior, and it can stimulate employee voice behavior.

However, the effect of “resource” on employee voice behavior has not been fully considered in previous research. Regarding the relationship between the helpful behavior of the leader and the voice of the employee, there are some controversies that need clarification. First, employees experience pressure and then exhibit reduced voice behavior when they are under resource danger from organizational, social, and workplace factors. Many workers in different contexts realize that managers’ response to voice behavior can be negative, especially in societies like China. Voice behavior is a behavior that defies status quo and can be interpreted in Eastern societies as a disregard for the manager’s authority or criticism of his/her performance. Asian countries are generally considered high in terms of power distance and collective dimensions, which means that hierarchy and power have a lot to do with the expectation of a manager’s response to voice behavior (Yang et al., 2021b). Some employees are skeptical of their managers’ patience and generosity. Employees who speak up frequently may be concerned about the impact on their professional image, relationships, or future career development (Hsiung, 2012). The second aspect of confusion is that the helping behavior that a manager may practice is an indicator of the personal citizenship behavior that supports and maintains the status quo, while the voice behavior mimics the personal citizenship behavior that changes, threatens, or disrupts the status quo (Grant, 2013).

The issue of how to supply and what kind of resources to enhance employee voice behavior has not been resolved by existing research. Therefore, the first research question of this study is, “How does supervisor helping behavior affect employee voice behavior?” Studies have mainly focused on inter-individual factors with little attention paid to intra-individual factors. But Liu et al. (2017) discussed stable intra-individual differences as well as the existence of dynamic trends, which provide a novel idea for voice behavior research.

Although supervisor helping behavior can assist employees in gaining access to resources directly, the internal “black box” mechanism underlying supervisor helping behavior and employee voice behavior is unclear and only a few empirical studies have investigated this issue. In the present research on supervisor support and employee voice, as recommended by Bergeron and Thompson (2020), it is vital to examine pertinent mediating variables, particularly to examine people’s internal psychological processes. Supervisor’s behavior has a significant impact on employee’s attitude, work behavior, and physical and mental health, and the inner psychology of employees inspired by leadership may be an important influencing factor (Kleine et al., 2019). Spreitzer et al. (2005) developed the concept of “thriving at work,” which means that employees experience a state of vitality and learning at work. Therefore, the second question we proposed is, “What is the internal mechanism between supervisor helping behavior and employees’ voice behavior?” We argue that supervisors can promote employees’ autonomy, ability, and belongingness by providing assistance, thereby affecting employees’ thriving at work. Accordingly, employees’ psychological security is improved in the state of thriving at work, making them more willing to take risks and share advice with superiors (Yang et al., 2021a).

Additionally, few studies have discussed contextual factors that influence the “antecedents-thriving at work” process (Shahid et al., 2020). Based on the conservation of resource theory, the interconversion of resources between individuals and the environment makes employees feel valued by meeting their individual needs (Dong et al., 2020). Support from supervisors can improve employees’ psychological satisfaction and motivate their work vigor and learning enthusiasm. But the relationship between voice behavior and resources is not static, and the possible boundary conditions between them need to be further discussed and analyzed. Therefore, the third question is, “What are the boundary conditions for supervisor helping behavior affecting thriving at work?” We propose that higher psychological availability can enhance employees’ sense of security, influencing the relationship between supervisor helping behavior and employees’ thriving at work (Li and Tong, 2021).

This study provides a more detailed account of the influence of leadership behavior on employee voice behavior than previous studies. Despite the fact that the notion of helpful behavior has become one of the most fascinating topics in organizational research in recent years, research on the influence of leadership behavior on it is still in its early stages of conceptual growth and requires empirical research (Bergeron and Thompson, 2020).

By developing a cross-hierarchical model, this study contributes to the literature on leadership and voice behavior in several ways. First, based on the conservation of resources theory, this study adopts the experience sampling method (ESM) to investigate the impact of supervisor helping behavior on employee voice behavior from a dynamic perspective. This study enriches the existing research findings of supervisor support and servant leadership. Second, it reveals the internal mechanism of thriving at work and explores the individual influence of daily supervisor helping behavior on employee voice behavior. Bergeron and Thompson (2020) called for further exploration of other mediating variables in future research, and this study supplemented relevant findings on the mediating influence between supervisor helping behavior and employee voice behavior. Third, in response to the call of Halbesleben et al. (2014), this study explored the influence of the interaction between internal and external resources on the working state and behavior of employees. We argue that leadership helping behavior can provide external resources, and psychological availability as an important internal resource also plays a very important role. This empirical research introduces psychological availability and analyzes the boundary conditions of supervisor helping behavior as they related to thriving at work.

## Literature review and hypothesis

### Conservation of resource theory

This study adopts the conservation of resource theory to investigate the effect of psychological availability, supervisor helping behavior, thriving at work, and employee voice behavior. Conservation of resource theory provides a theoretical framework that employees have the motivation to acquire and safeguard their resources (Hobfoll, 1989). Hobfoll argued that objects that are highly valued by individuals help them achieve goals, such as personal characteristics, conditions, and energies. According to the conservation of resource theory, when employees face the threat of resource loss, they will strive to preserve and acquire the valuable ones because the resource is the most important (Zhao and Guo, 2019). At the same time, employees will be positive and less anxious if they are satisfied with their resources.

Conservation of resource theory provides the idea that the leader’s helping behavior can give followers the impression that they are providing them with the necessary resources. Individual tension and stress can be relieved by this illusion of extra resources (Hobfoll et al., 2018). Employees in this position will comply with leaders’ requests for assistance, put in a lot of effort, improve their rapport with them, and aggressively build and preserve their existing resource reserve in order to deal with the eventuality of resource loss in the future (Hobfoll et al., 2018).

### Supervisor helping behavior and employee voice behavior

Employee cooperation and supervisor helping behavior are increasingly important in modern organizations (Zhang et al., 2019). Employees are concerned with maximizing their interests. Supervisors can also influence employees’ cognition and reduce self-interested behaviors by setting an example of helping behaviors (Frazier and Tupper, 2018). From the perspective of conservation of resource theory, supervisors will help employees by reserving their existing resources, actively constructing and protecting social relationships, and through such resource investment, acquire new resources and cope with possible future stressful situations (Hobfoll et al., 2018). As a result, supervisor helping behavior belongs to both civic behavior and organizational behavior, and it can benefit both partial individuals and the entire organization (Spitzmuller and Dyne, 2013). Research has shown that supervisor helping behavior is essentially a subordinate and cooperative relationship that can increase personal welfare and mutual trust, and the organization can benefit from the supervisor helping behavior (Jia et al., 2021).

Voice behavior is a constructive change-oriented behavior used to improve the organizational environment (Townsend et al., 2020). Voice behavior, based on changing the status quo, and sometimes even threatening the organization norms and supervisor authority, is regarded as a challenging organizational citizenship behavior. Studies have been primarily based on social identity theory and cognitive-affective personality system theory to investigate the factors that influence voice behavior. They mainly concentrated on personal characteristics, organizational context, and supervisor behavior (Mowbray et al., 2015), including the use of a hierarchical linear model to investigate the influence of target-oriented supervisors on employee voice behavior (Zhu and Akhtar, 2019).

Based on the conservation of resource theory, this study argues that supervisors can provide employees with material, position, time, knowledge, and other helpful resources (Hobfoll et al., 2018). When employees perceive that they can receive external resources from their supervisors, they will reduce resource anxiety and tension. Additionally, employees will take the initiative to maintain good rapport with others when they expect to be rewarded (Cortez and Johnston, 2020). Simultaneously, the reciprocity principle states that when employees perceive benefits from supervisors, they will work harder to reward their boss (Moore et al., 2020). The work-related resources provided by the organization will affect employees’ psychological perceptions. As a result, the supervisor will set a good example by providing adequate care and respect to employees to encourage extra-role behavior and improve the overall competitiveness of the organization. In return and response, the voice behavior will point out organizational problems. Studies have also shown that supervisor helping behavior can significantly improve employees’ voice behavior (Detert and Burris, 2007). On this basis, we propose:

*Hypothesis 1*: Supervisor helping behavior is positively related to employee voice behavior.

### Mediating effect of thriving at work

Thriving at work is a temporary psychological state of employees. When employees are thriving at work, they can feel vitality and experience the learning emotion (Spreitzer et al., 2005). “Vitality” refers to energetic positive emotions, whereas “learning emotion” refers to a feeling of the ability to acquire and apply knowledge. This study argues that supervisor helping behavior can improve the employees’ thriving at work. First, supervisors who lead by example will influence employees’ cognition through their role models, and supervisors improve employees’ work autonomy by caring for them (Tu et al., 2017). Second, supervisor helping behavior can stimulate employees’ extra-role behavior (Zhang and Xie, 2017). When improving enterprise management performance, employees can also enhance their work capacity. In addition, by assisting and interacting with employees, supervisors can create an environment of mutual trust so that employees can gain a sense of psychological security and belongingness (Loi et al., 2011).

Supervisor helping behavior is a beneficial context for employees to enhance their thriving at work. Based on the dynamic perspective of conservation of resource theory, thriving motivates employees to voice to achieve resource gain spirals. Employees will perceive their progress and energy gradually when their work autonomy and belongingness are satisfied, thereby maintaining work vitality and novelty. They can not only realize their potential but also improve their competence accordingly. This dynamic improvement in work efficiency is the consequence of employees’ thriving at work (Shahid et al., 2020). When employees are thriving at work, they can focus on their work without worrying about negative influences, giving them psychological security (Edmondson, 1999). Employees giving advice may touch the “cordon” due to the risks of voice behavior (Son, 2019). As a result, implementing voice behavior should be a trade-off comprehensively. Positive emotions can help employees broaden the range of pros and cons to accelerate decision-making efficiency (Liao et al., 2019). Psychological security can provide employees with positive emotions that encourage them to engage in challenging or risky behaviors, so employees who are in thriving at work tend to make voice behavior choices (Sonnentag and Grant, 2012). On this basis, we propose:

*Hypothesis 2*: Thriving at work mediates the relationship between supervisor helping behavior and employee voice behavior.

### Moderated effect of psychological availability

When analyzing the process of preserving and acquiring individual resources, the early COR theory gives little emphasis to the interactions between various resources. Hobfoll (2012) made the observation that many resources are interconnected and have an impact on one another like a moving motorcade rather than existing alone. Additionally, the environment through which the vehicle travels plays a significant influence. More focus on the interactions between various resources and the impact of environmental factors is also recommended by Halbesleben et al. (2014). As a result, the possible impact of employees’ internal and external resources is the main focus of this study.

“Psychological availability” refers to the perception of various available resources at work, including physiological and emotional aspects, which belongs to employees’ internal emotion or internal resources (Qian et al., 2020). Conservation of resource theory argues that resources such as materialism, personality, and energy are interconverted between individuals and the environment (Hobfoll, 1989). Employees will meet their needs when resources are obtained from above. Individually, supervisor helping behavior will provide employees with external resources such as cars, housing, and rights to decrease work pressure (Frazier and Tupper, 2018).

Psychological availability is a kind of internal resources for employees which interacts with the contextual resources to serve the employees. With a high level of psychological availability, employees will have a high level of psychological security, and their work vitality will increase correspondingly (Li and Tong, 2021). They will actively learn to demonstrate their responsible attitude and work capacity for the enterprise. As a result, the thriving at work will be enhanced (Zeng et al., 2020). On this basis, we propose:

*Hypothesis 3*: Psychological availability moderate the relationship between supervisor helping behavior and employees’ thriving at work. The higher the level of psychological availability, the stronger the effect of supervisor helping behavior on employees’ thriving at work.

In addition to the study mentioned above, we also investigate the patterns of moderated mediation across the entire voice behavior process. The relationship between supervisor helping behavior and employee voice behavior can specifically be transmitted by employees’ thriving at work; however, this transmission impact also depends on the psychological availability of the employees. According to May et al. (2004), psychological availability as a state can help people decide whether or not to engage in organizational activities. In essence, psychological availability partly reflects employees’ confidence in their work. If employees have higher psychological availability and access to the work necessary resources, they will be better prepared for unknown work challenges. As a result, they are more motivated to do something outside of their job, such as voice behavior (Binyamin and Carmeli, 2010). Furthermore, studies have found that employees with high psychological availability contribute more to their work and innovate more (Wang et al., 2021a). They are more willing to participate in innovation work, whether they complete it alone or in collaboration with others (Byrne et al., 2016). In such a work environment, employees’ innovation can be further improved, including their thriving at work (Wang et al., 2021b). Therefore, the higher vitality and enthusiasm for the job can improve the thriving at work. Based on this, we propose:

*Hypothesis 4*: Psychological availability moderates the indirect influence of supervisor helping behavior on employee voice behavior through employees’ thriving at work. This indirect relationship is observed when psychological availability is high.

**Figure 1:**
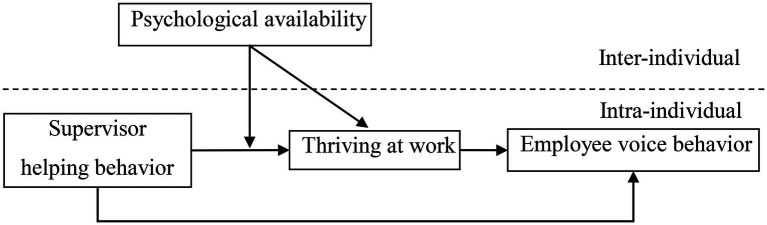
Theoretical model.

Based on the preceding analysis, this study developed a cross-hierarchical model depicted in Figure 1.

## Design

### Method

This study adopts the ESM to investigate the influence of supervisor helping behavior on employee voice behavior from a dynamic perspective. Before collecting data, the following sample selection criteria are determined: working hours were limited to 5 days of full-time employment and no fewer than 40 h per week. To lessen the impact of time differences, the workplace was located in China. We randomly selected 113 enterprise candidates, informed them of the research goal and procedure, and inquired about their willingness to participate in the study. Eventually, 76 people agreed to participate in the survey.

The data collecting process lasted for 6 days. The research group delivered a personal trait questionnaire (including questionnaire code, gender, education level, age, and psychological availability) to the participants on the first morning of the experiment (Sunday). They needed to complete the questionnaire on that day. For the next 5 working days, the research group distributes the questionnaires at 11 a.m (measuring supervisor helping behavior) and 6 p.m (measuring thriving at work and voice behavior). In addition, participants need to complete the questionnaire within 1 h. Only two participants were eliminated for failing to complete the survey on time, resulting in a total of 74 participants who completed the assignment. The effective recovery rate was 97.36%.

In this survey, 41 people were female with a proportion of 55.4%. In terms of the education level, five college graduates and lower accounted for a proportion of 6.8%; 51 undergraduates with a proportion of 68.9%; and 18 graduates and above had a proportion of 24.3%. The average age was 29 years (standard deviation = ± 4.76). The participants are mainly from seven different industries, such as financial, information technology services, and manufacturing.

### Procedure

The scales used in this study were derived from top international journals and used the “back-to-back” translation into Chinese. Due to the pressure on employees to complete the forms every day, the original scale items were considerably altered and removed in this study. The scales use a 5-point Likert scale, ranging from “1 = strongly disagree” to “5 = strongly agree.”

Supervisor helping behavior adopted the scale developed by Farh et al. (1997), using statements such as “Today, my leaders are willing to help me solve the problems in my work” (*α* = 0.80). Employee voice behavior was measured using the scale developed by Dyne and Lepine (1998), which includes statements like, “Today I make suggestions on issues that affect work efficiency” (*α* = 0.87). Thriving at work adopted the scale developed by Porath et al. (2012), including statements such as, “Today, I find myself constantly improving,” and, “Today, I feel vibrant” (*α* = 0.87). Psychological availability was measured using a scale developed by Byrne et al. (2016), such as, “Recently, I was emotionally ready to meet job requirements” (*α* = 0.96). It asked participants to answer based on their work experience over the past 3 months.

In addition, based on previous research, this study selected control variables that may affect voice behavior, including gender (0 = male, 1 = female), education level (1 = college and below, 2 = undergraduate, 3 = postgraduate, and above), and age. Following the suggestion of Aguinis and Vandenberg (2014), controlled variables and uncontrolled variables were analyzed respectively, and the results were the same. And removing control variables from the equation model does not change the interpretation of the results.

## Empirical analysis

### Confirmatory factor analysis

The data of this study were derived from a self-rating scale for multi-hierarchical confirmatory factor analysis with Mplus 7.0. Table 1 shows that the theoretical four-factor model has a better model fit ( $\chi^{2}$ (21) = 49.51, CFI = 0.99, TLI = 0.98, RMSEA = 0.06) compared to other models, indicating that the questionnaire used in this study has a good discrimination, and the common method variance will not affect the results.

**Table 1:** Confirmatory factor analysis.

	Variables	*x* ^2^	*df*	△*x*^2^	CFI	TLI	RMSEA	RMR
Four-factor model	LH, TW, V, PS	49.51	21		0.99	0.98	0.06	0.03
Three-factor model 1	LH + TW, V, PS	207.81	23	158.31^**^	0.93	0.89	0.15	0.13
Three-factor model 2	LH + V, TW, PS	206.10	23	156.60^**^	0.93	0.89	0.15	0.09
Three-factor model 3	V + TW, LH, PS	168.97	23	119.47^**^	0.95	0.91	0.13	0.12
Two-factor model	V + TW + LH, PS	322.97	24	273.47^**^	0.89	0.82	0.18	0.14

LH, leadership help behavior;

TW, work prosperity;

V, voice advice behavior; and

PS, psychological availability.

** *p* < 0.01.

### Descriptive statistics and correlation

The descriptive statistics of the intra-individual and inter-individual variables are shown in Table 2. At the intra-individual level, there is a significant positive correlation between supervisor helping behavior, voice behavior (*r* = 0.37, *p* < 0.01), and thriving at work (*r* = 0.38, *p* < 0.01). There is a significant positive correlation between thriving at work and voice behavior (*r* = 0.31, *p* < 0.01). At the inter-individual level, there is a significant positive correlation between psychological availability and education level (*r* = 0.19, *p* < 0.01).

**Table 2:** Descriptive statistics and correlation.

Intra-individual	mean	SD	% of Variance	1	2	3	
1. Voice behavior	3.00	1.13	0.37	(0.90)			
2. Thriving at work	3.27	0.95	0.36	0.31^**^	(0.84)		
3. Supervisor helping behavior	2.83	1.10	0.38	0.37^**^	0.38^**^	(0.90)	
**Inter-individual**	**Mean**	**SD**		**1**	**2**	**3**	**4**
1. Gender	–	–		–			
2. Age	29.08	4.76		0.04	–		
3. Education level	–	–		0.09	−0.23^**^	–	
4. Psychological availability	3.80	0.66		−0.14^**^	−0.13^*^	0.19^**^	(0.95)

* *p* < 0.05;

** *p* < 0.01.

### Hypothesis test results

**Figure 2:**
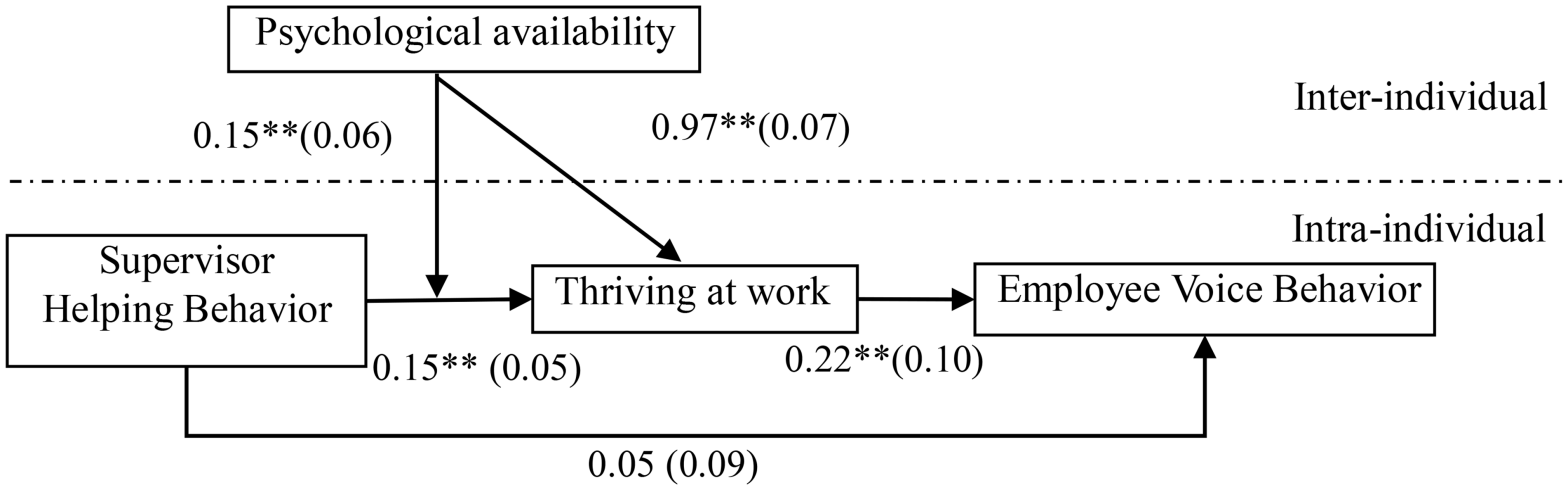
The results of cross-hierarchical equations analysis. **p < 0.01.

This study adopts Mplus 7.0 to test the cross-hierarchical mode. The intra-individual variables (i.e., voice behavior, thriving at work, and supervisor helping behavior) adopt a group mean with mean centering, and the inter-individual moderator variable (psychological availability) adopts a total mean with mean centering. The first step before performing cross-hierarchical analysis is to calculate the intra-individual level percentages of variation for these three variables. The percentages of supervisor helping behavior, thriving at work, and voice behavior were 38.1, 35.6, and 37%, respectively, indicating that the data were suitable for a cross-hierarchical analysis. According to Roesch et al. (2010), this study uses a cross-hierarchical equation model for hypothesis testing. Figure 2 depicts the result of cross-hierarchical equations analysis. After controlling the variables, supervisor helping behavior has no significant effect on employee voice behavior (*γ* = 0.05, not significant).

Supervisor helping behavior had a significant positive effect on thriving at work (*γ* = 0.05, *p* < 0.01), and thriving at work had a significant positive effect on employee voice behavior (*γ* = 0.22, *p* < 0.01). To further test the mediating effect of thriving at work between supervisor helping behavior and voice behavior, this study adopts Monte Carlo’s bootstrapping test, and the results are shown in Table 3. The direct effect of supervisor helping behavior on voice behavior is 0.05, with a 95% confidence interval of [−0.12, 0.23]. The interval contains a value of zero, indicating that the direct effect is supported. The indirect effect is 0.03, with a 95% confidence interval of [0.01, 0.07]. The interval did not contain 0, indicating that the mediating effect is supported. As a result, Hypothesis 2 is supported.

**Table 3:** Simple slope analysis and moderated mediating effect test.

Simple slope analysis	Value	SD	95% LLCI	95% ULCI
Low psychological availability	0.01	0.02	−0.02	0.05
High psychological availability	0.06	0.03	0.01	0.12
Difference	0.05	0.03	0.01	0.10
**Moderated mediating effect test**	**Indirect effect**	**SD**	**95% LLCI**	**95% ULCI**
Low psychological availability	0.05	0.06	−0.07	0.17
High psychological availability	0.25	0.06	0.12	0.38
Difference	0.20	0.08	0.04	0.36
**Mediating effect test**	**Value**	**SD**	**95% LLCI**	**95% ULCI**
Direct effect	0.05	0.09	−0.12	0.23
Indirect effect	0.03	0.02	0.01	0.07

**Figure 3:**
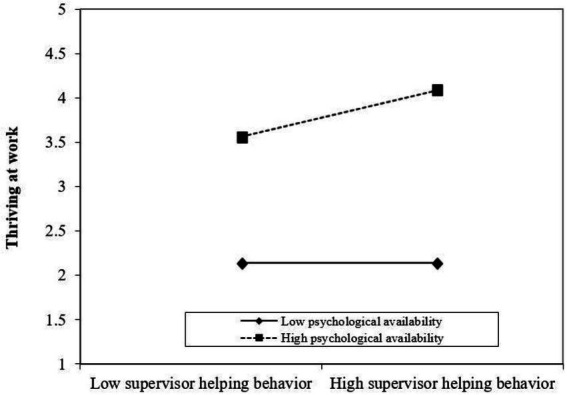
The moderate effect of psychological availability.

To further test the moderating effect of psychological availability, this study analyses the slope using Monte Carlos’s bootstrapping test. The results are shown in Table 3. Under the situation of low psychological availability, the effect of supervisor helping behavior on thriving at work is 0.01, and the 95% confidence interval is [−0.02, 0.05]. The interval contains 0, indicating no significant effect of supervisor helping behavior on thriving at work. Under the situation of high psychological availability, the effect of supervisor helping behavior on thriving at work is 0.06, and the 95% confidence interval is [0.01, 0.12]. The interval did not contain 0, indicating a significant effect of supervisor helping behavior on thriving at work. This study further tested the difference in the slopes under the two conditions. The difference is 0.05. The 95% confidence interval is [0.01, 0.10], excluding 0, indicating that the slopes under the two conditions were significantly different. The moderating effect of psychological availability is significant between supervisor helping behavior and thriving at work. Thus, Hypothesis 3 is supported (Figure 3).

There was a significant cross-hierarchical interaction between thriving at work and psychological availability (*γ* = 0.97, *p* < 0.01). To further test the moderated mediating effect, this study adopts Monte Carlo’s bootstrapping test. The results are shown in Table 3. In the situation of low psychological availability, the mediating effect of thriving at work between supervisor helping behavior and voice behavior is 0.05. And the 95% confidence interval is [−0.07, 0.17], which contains 0 within the interval. As a result, it has no significant mediating effect. However, in the situation of high psychological availability, the mediating effect of thriving at work between supervisor helping behavior and voice behavior was 0.25, and the 95% confidence interval was [0.12, 0.38], excluding 0 in the interval. As a result, the mediating effect is significant. The study further tested the difference of the mediating effect between the two situations. The difference is 0.20, and the 95% confidence interval was [0.04, 0.36]. Furthermore, the interval does not contain 0, indicating that the difference of the mediating effect is significant. As a result, Hypothesis 4 is supported.

## Discussion

### Results

Based on conservation of resource theory, the ESM is adopted in this study to investigate the influence of supervisor helping behavior on employee voice behavior from a dynamic perspective. This study proposes a cross-hierarchical model that includes supervisor helping behavior, thriving at work, psychological availability, and voice behavior. The data-collecting process lasted for 6 days. The results show that supervisor helping behavior cannot directly promote employees’ voice behavior, but thriving at work mediates supervisor helping behavior and employees’ voice behavior. In addition, psychological availability moderates the relationship between supervisor helping behavior and thriving at work. The results show that the higher the level of psychological availability, the stronger the effect of supervisor helping behavior on employees’ thriving at work. And finally, under the moderation of psychological availability, supervisor helping behavior on voice behavior has a more significant effect through thriving at work.

The results reveal the dynamic promotion effect of supervisor helping behavior on voice behavior at the intra-individual level. Psychological availability is also considered at the inter-individual level. The higher the level of psychological availability, the more likely it is to stimulate voice behavior through the resource conversion within the individual. From a dynamic perspective, this study clarifies the relationship between supervisor helping behavior and voice behavior, and it contributes to organizational human resource management.

### Theoretical implication

First, the ESM is adopted in this study to investigate the influence of supervisor helping behavior on employee voice behavior with a dynamic perspective from inter-individual to intra-individual. This study enriches the related literature on supervisor support and employee voice behavior. Previous studies mainly discussed the importance of voice behavior, seeking to identify their consequences (Chou and Barron, 2016). Although some studies have investigated the influence of leadership style on employees’ voice behavior, most of these studies start from the inter-individual level or the organizational level. For example, Li and Sun (2015) found that supervisor authoritarian leadership has a negative effect on employees’ voice behavior, whereas supervisor authoritarian leadership has a cascading effect on employee voice behavior. These effects can be strengthened or weakened in different conditions (i.e., leader identification and power distance orientation). Based on 359 samples from five-star hotels in Taiwan, Zhuang et al. (2021) investigated the effects of supervisors’ moral leadership, benevolent leadership, and authoritarian leadership on employees’ voice behavior. With a sample of 527 Korean employees from 35 companies, Lee et al. (2021) investigated the effect of positive feedback from supervisors on subordinates’ voice behavior. Although these studies investigate the causes of voice behavior, they ignored the exploration from the intra-individual level. Using the empirical sampling method, Liu et al. (2017) and Starzyk et al. (2018) discovered stable intra-individual differences in voice behavior. These authors demonstrate a possible dynamic trend in voice behavior, providing a new perspective on employees’ voice behavior. However, only three studies have explored voice behavior with the empirical sampling method (Liu et al., 2017; Starzyk et al., 2018; Zhang et al., 2019). This study adopts the empirical sampling method to conduct a cross-hierarchical analysis of voice behavior which enriched the literature on voice behavior.

Second, this paper enriches the relevant research on supervisor helping behavior through introducing thriving at work as an antecedent variable. Recently, scholars have begun to pay attention to helping behavior, especially the helping behavior between employees from the team level (Liang et al., 2015; Jin et al., 2021). However, there are few studies on supervisor helping behavior, which is reflected by giving employees psychological and material support, such as care, respect, and material resources. Spreitzer et al. (2005) mentioned the use of models for social insets to investigate thriving at work in which the model mentions departmental contextual factors and work resources. The way to complete the work, knowledge, and emotion obtained by employees seems to affect the perceived vitality of employees (Kark and Carmeli, 2009). Based on the conservation of resource theory, this paper investigates the resource conversion between supervisor helping behavior and employees’ thriving at work and theoretically enriches the literature on supervisor helping behavior and thriving at work.

Third, most of the existing studies on servant leadership and employee voice behavior have reached significant conclusions. In the exploration of intermediary mechanism, scholars mainly analyzed psychological safety, job engagement, organizational commitment, and organizational identification (Chughtai, and Ali, 2016; Lapointe and Vandenberghe, 2018; Song et al., 2021). This study focuses on the concept of supervisor helping behavior, which is close to that of servant leadership. But one is leadership behavior and other one is leadership characteristics. On the whole, this study expands the research framework of servant leadership and provides new ideas for related research. Future scholars, for example, can pay attention to the mediation role of thriving at work in the effect of servant leadership and employee voice behavior.

In addition, this study investigates the psychological availability at the intra-individual level, which further analyzes the moderate role of psychological availability between supervisor helping behavior and thriving at work, and the mediating effect of thriving at work between supervisor helping behavior and voice behavior. Previous studies on psychological availability mainly regard it as an intermediary mechanism while few studies regard it as a situational variable (Wang et al., 2021a). For example, through psychological availability, Qian et al. (2020) investigated the indirect effect of supervisor guidance behavior on employee knowledge sharing. Geldenhuys and Łaba (2018) found that daily psychological availability has a mediating effect between positive work–family interaction and daily work engagement. This study further expands the relevant theoretical research on psychological availability and explores psychological availability as a moderate role from a dynamic perspective. It enriches the literature on psychological availability on the cross-hierarchical influence between supervisor helping behavior and employees’ voice behavior.

### Practical implications

Our research brings significant implications for practice. First, the results show that supervisor helping behavior promotes employee voice behavior through resource conversion. Therefore, supervisors should attach importance to assisting employees in their daily work, including material resource support and psychological encouragement. The helping behavior is essentially a cooperative relationship, which can enhance the trust among the members (Glomb et al., 2011). To maintain this trust, supervisors should treat employees with full respect so that employees can feel the benefits given by others and take the initiative to give back to the enterprise, such as through voice behavior. Voice behavior spontaneously based on the initiative can promote the innovative development of the organization.

Second, this study focuses on the intra-individual variables, introduces the mediating variable of thriving at work, and finds that thriving at work plays a conductive role between supervisor helping behavior and employee voice behavior. Because thriving at work is affected by the work situation and resources, supervisors should set a good example to influence employees’ cognition, give them enough care, and strive to create a mutual trust environment, which can help employees gain psychological security (Sonnentag and Grant, 2012). When employees feel psychological security, it is easier for resource conversion and stays in a state of thriving. Employees in thriving at work are more willing to actively perceive work vitality and learning changes to determine whether and how they can take actions at work. This self-adaption ability helps employees take the initiative, such as voice behavior, to promote the organization’s development.

Finally, psychological availability has a positive moderate effect on the thriving at work, which means that employees with high psychological availability are more willing to act voice behavior through resource conversion. Enterprises with positive culture as strategic orientation will often gain competitive advantages (Fan and Hou, 2022). Therefore, enterprises should improve their psychological availability by paying attention to what employees need. Supervisors can create harmonious working environments by actively asking employees what they desire and encouraging them to maintain a good relationship. At the same time, supervisors can actively encourage employees to participate in sports activities to improve their physical fitness and give employees some guidance to improve their vocational skills and regularly arrange some career planning courses for them.

### Limitations and future research

As with any empirical study, ours has several limitations that point to avenues to future research. First, although this study adopts the multi-point empirical sampling method, the relationship between the variables can be tested to a certain extent. The influence of irrelevant factors can be excluded by experiment or cross-lag analysis in the future to determine the causal relationship between the core variables.

Second, the scales we used in this study were self-reported; it may exist with potential common method variance. Although common method variance is controlled through rigorous design and statistics, it is still necessary to add other evaluation data in subsequent studies to ensure rigor and stability (Fuller et al., 2016).

Third, some scholars pointed out that supervisors can influence employees’ psychological security and interfere with their voice behavior (Holley et al., 2018). Therefore, when investigating the influence of supervisors on voice behavior, psychological security can also become a mediating factor. This study only explored the mediating role of thriving at work at the intra-individual level and not the role of other mediators such as psychological security. Future research can take psychological security into account and analyze its impact on voice behavior at the intra-individual level.

## Conclusion

Using the experience sampling method, this study discusses the dynamic mechanism of supervisor helping behavior and employee voice behavior as well as the boundary conditions. In addition, based on resource conservation theory, this study developed a hierarchical model of supervisor helping behavior, thriving at work, psychological availability, and employee voice behavior. It contributes to answer how and when employee thriving at work and voice behavior are shaped by supervisor helping behavior.

## Data availability statement

The original contributions presented in the study are included in the article/supplementary material, further inquiries can be directed to the corresponding author.

## Ethics statement

Ethical review and approval was not required for the study on human participants in accordance with the local legislation and institutional requirements. Written informed consent for participation was not required for this study in accordance with the national legislation and the institutional requirements.

## Author contributions

PF drafted the manuscript and contributed to the interpretation of the results. YL contributed to the English version of the manuscript. HL collected the data and carried out analyses. MH contributed to the important comments, critically revised the manuscript, and provided the necessary resources for this research. All authors contributed to the article and approved the submitted version.

## Funding

This study was supported by the Fund of Humanities and Social Sciences, Ministry of Education (19YJC630032), Shanghai Philosophy and Social Science Planning Project (2018EGL009), and research project of Shanghai International Studies University (2018114036).

## Conflict of interest

HL was employed by the company NIO Inc.

The remaining authors declare that the research was conducted in the absence of any commercial or financial relationships that could be construed as a potential conflict of interest.

## Publisher’s note

All claims expressed in this article are solely those of the authors and do not necessarily represent those of their affiliated organizations, or those of the publisher, the editors and the reviewers. Any product that may be evaluated in this article, or claim that may be made by its manufacturer, is not guaranteed or endorsed by the publisher.
